# Expression of platelet-derived endothelial cell growth factor (PD-ECGF) and its mRNA in uterine cervical cancers

**DOI:** 10.1038/sj.bjc.6690200

**Published:** 1999-03

**Authors:** J Fujimoto, H Sakaguchi, R Hirose, S Ichigo, T Tamaya

**Affiliations:** Department of Obstetrics and Gynaecology, Gifu University School of Medicine, 40 Tsukasa-machi, Gifu City 500-8705, Japan

**Keywords:** platelet-derived endothelial cell growth factor, angiogenesis, uterine cervical cancer

## Abstract

Angiogenesis contributes to the growth and secondary spreading of solid tumours. Platelet-derived endothelial cell growth factor (PD-ECGF) is identified as such an angiogenic factor. In the present study, the prognosis of the patients with high PD-ECGF uterine cervical cancers was worse than those with low PD-ECGF cancers, and PD-ECGF expression correlated with cellular proliferation and with vascular density and venous invasion in uterine cervical cancers. Therefore, PD-ECGF might contribute to the growth of uterine cervical cancers via angiogenesis related to vascular spreading. Furthermore, PD-ECGF and its mRNA had a wide range and were highly expressed in uterine cervical cancers, especially squamous cell carcinoma, regardless of clinical stage. Therefore, PD-ECGF in uterine cervical cancers might play a role of basic angiogenesis in all processes of advancing of uterine cervical cancers. This indicates that 5′-deoxy-5-fluorouridine might be highly effective in squamous cell carcinoma of the cervix, which possesses a high activity of thymidine phosphorylase to convert 5′-deoxy-5-fluorouridine to 5-fluorouracil, and that some angiogenic inhibitors of new capillary formation might be effective in the inhibition of tumour growth and spreading associated with angiogenesis. © 1999 Cancer Research Campaign


*British Journal of Cancer* (1999) **79**(7/8), 1249–1254
© 1999 Cancer Research Campaign
Article no. bjoc.1998.0200

### Expression of platelet-derived endothelial cell growth factor (PD-ECGF) and its mRNA in uterine cervical cancers

**J Fujimoto, H Sakaguchi, R Hirose, S Ichigo and T Tamaya**

Department of Obstetrics and Gynaecology, Gifu University School of Medicine, 40 Tsukasa-machi, Gifu City 500-8705, Japan

**Summary** Angiogenesis contributes to the growth and secondary spreading of solid tumours. Platelet-derived endothelial cell growth factor (PD-ECGF) is identified as such an angiogenic factor. In the present study, the prognosis of the patients with high PD-ECGF uterine cervical cancers was worse than those with low PD-ECGF cancers, and PD-ECGF expression correlated with cellular proliferation and with vascular density and venous invasion in uterine cervical cancers. Therefore, PD-ECGF might contribute to the growth of uterine cervical cancers via angiogenesis related to vascular spreading. Furthermore, PD-ECGF and its mRNA had a wide range and were highly expressed in uterine cervical cancers, especially squamous cell carcinoma, regardless of clinical stage. Therefore, PD-ECGF in uterine cervical cancers might play a role of basic angiogenesis in all processes of advancing of uterine cervical cancers. This indicates that 5′-deoxy-5-fluorouridine might be highly effective in squamous cell carcinoma of the cervix, which possesses a high activity of thymidine phosphorylase to convert 5′-deoxy-5-fluorouridine to 5-fluorouracil, and that some angiogenic inhibitors of new capillary formation might be effective in the inhibition of tumour growth and spreading associated with angiogenesis.

**Keywords:** platelet-derived endothelial cell growth factor; angiogenesis; uterine cervical cancer

Angiogenesis is essential for nutrition and growth of solid tumours greater than 2 mm in diameter (Folkman, 1985). Unorganized basement membrane of new capillary endothelial cells allows intravasation of tumour cells, and a high density of microvessels in tumours is associated with their expansion and invasiveness (Srivastava et al, 1988; Weidner et al, 1991, 1993; Macchiarini et al, 1992; Wakui et al, 1992). Angiogenesis consists of the following steps: dissolution of basement membrane by proteases released from tumour or host cells which have been activated by tumour-derived angiogenic factors, migration and proliferation of endothelial cells, and capillary tube formation (Folkman and Haudenschild, 1980).

The following angiogenic factors have been identified: basic fibroblast growth factor (FGF), vascular endothelial growth factor (VEGF), placenta growth factor (PlGF), epidermal growth factor (EGF), transforming growth factor (TGF)-α, TGF-β, platelet-derived growth factor (PD-GF), platelet-derived endothelial cell growth factor (PD-ECGF), hepatocyte growth factor (HGF), tumour necrosis factor (TNF)-α, pleiotropin, proliferin, angiogenin, oestradiol, interleukin (IL)-8, etc. Main angiogenic factors induced from tumour cells are basic FGF, VEGF, PD-ECGF and IL-8. PD-ECGF was cloned as a novel angiogenic factor (45 kDa polypeptide) from human platelets (Ishikawa et al, 1989). Thereafter, PD-ECGF was completely identified with thymidine phosphorylase (TP) (Furukawa et al, 1992; Sumizawa et al, 1993). PD-ECGF/TP does not stimulate the growth of endothelial cells but rather chemotaxis of them, and induces angiogenesis in vivo with the activation of TP as an enzyme (Haraguchi et al, 1994; Miyadera et al, 1995). Among normal tissues, PD-ECGF is expressed in lymph nodes, peripheral lymphocytes, spleen, lung, liver, placenta (Yoshimura et al, 1990) and uterine endometrium (Fujimoto et al, 1998*a*; Fujimoto et al, in press). Among solid tumours, PD-ECGF is expressed in malignant gliomas, thyroid tumours, cancers of the breast, oesophagus, stomach, colon, pancreas, gall bladder, kidney, bladder, lung, uterine cervix (Yoshimura et al, 1990), uterine endometrium (Fujimoto et al, 1998*c*) and ovary (Fujimoto et al, 1998*b*).

To know the potential of growth, invasion and metastasis of uterine cervical cancer associated with angiogenesis, we studied the correlation of PD-ECGF expression with patients' prognosis, cellular proliferation, vascular density and venous invasion, and the expressions of PD-ECGF and its mRNA related to histopathological types and clinical stages of uterine cervical cancers.

*Received 19 November 1997*
*Revised 27 May 1998*
*Accepted 2 June 1998*

*Correspondence to:* J Fujimoto

### MATERIALS AND METHODS

### Patients

Agreements for the following studies were obtained from all patients and the Research Committee for Human Subjects, Gifu University School of Medicine. One hundred and forty patients ranging from 36 to 81 years of age underwent hysterectomy for uterine leiomyoma, or hysterectomy or biopsy for cervical cancer at the Department of Obstetrics and Gynaecology, Gifu University School of Medicine, between January 1994 and October 1997. None of the patients had received any therapy. A part of each uterine cervical cancer and normal cervix as controls was obtained immediately after hysterectomy and was snap-frozen in liquid nitrogen to determine the levels of PD-ECGF and its mRNA expressions, and a neighbouring part of the tissues was submitted for histopathological study. The clinical stage of uterine cervical cancers was determined by the International Federation of Obstetrics and Gynaecology (FIGO) classification (FIGO News, 1989). Sixty-two patients underwent curative resection for uterine cervical cancer and were observed for a 24-month survival rate. For these patients, immunohistochemical staining for PD-ECGF, Ki-67 and factor VIII-related antigen was carried out to analyse PD-ECGF functions related to cellular proliferation and to microvessel density and venous invasion.

### Enzyme immunoassay for determination of human PD-ECGF antigen

All steps were carried out at 4°C. Tissues (wet weight 10–20 mg) were homogenized in HG buffer (5 mM tris-HCl, pH 7.4, 5 mM sodium chloride, 1 mM calcium chloride, 2 mM EGTA, 1 mM magnesium chloride, 2 mM dithiothreitol (DTT), 25 μg $ml^{-1}$ aprotinin, and 25 μg $ml^{-1}$ leupeptin) with a Polytron homogenizer (Kinematics, Luzern, Switzerland). This suspension was centrifuged in a microfuge at 12 000 r.p.m. for 3 min to obtain the supernatant. The protein concentration of samples was measured by the method of Bradford (1976) to standardize PD-ECGF antigen levels.

PD-ECGF antigen levels in the sample were determined by the sandwich enzyme immunoassay described by Nishida et al (1996). The levels of PD-ECGF were standardized with corresponding cellular protein concentrations.

### Immunohistochemistry

For formalin-fixed paraffin-embedded tissues, 4-μm sections were cut with a microtome and dried overnight at 37°C on a silanized slide (Dako, Carpinteria, USA). Samples were deparaffinized in xylene at room temperature for 80 min and washed with a graded ethanol/water mixture and then with distilled water. The samples for PD-ECGF were soaked in phosphate-buffered saline (PBS), those for Ki-67 were soaked in a citrate buffer and then autoclaved at 121°C for 10 min, and those for factor VIII-related antigen were treated with 0.3 μg $ml^{-1}$ trypsin in PBS at room temperature for 20 min. The protocol for a Dako LSAB2 Kit, Peroxidase (Dako) was followed for each sample. In the described procedures, mouse anti-human PD-ECGF antigen 654-1 [10 μg $ml^{-1}$, Nippon Roche, Kamakura, Japan (Nishida et al, 1996)], rabbit anti-human Ki-67 antigen (10 μg $ml^{-1}$, Dako), and rabbit anti-factor VIII-related antigen (Zymed, San Francisco, USA) were used at dilutions of 1:100, 1:50 and 1:2 respectively. The proliferating cell population was evaluated using the Ki-67 index (Nakano and Oka, 1993). Vascular density was evaluated with microvessel counting (Maeda et al, 1996).

### Reverse transcription polymerase chain reaction (RT-PCR) to amplify PD-ECGF mRNA

Total RNA was isolated from the cells by the acid guanidium thiocyanate–phenol–chloroform extraction method (Chomczynski and Sacchi, 1987). Total RNA (3 μg) was reverse transcribed with Moloney murine leukaemia virus reverse transcriptase (MMLV-RTase, 200 units, Gibco BRL, Gaithersburg, MD, USA) in a buffer of 20 mM tris-HCl, pH 8.4, 50 mM potassium chloride, 2.5 mM magnesium chloride, 0.1 mg $ml^{-1}$ bovine serum albumin, 10 mM DTT and 0.5 mM deoxynucleotides to generate cDNAs using random hexamer (50 ng, Gibco BRL) at 37°C for 60 min. The RT reaction mixture was heated at 94°C for 5 min to inactivate MMLV-RTase.

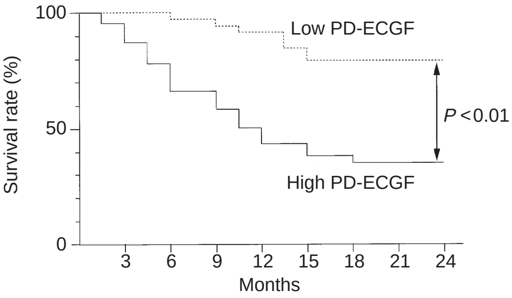

**Figure 1** Survival rate after curative resection for squamous cell carcinoma of the uterine cervix. Prognosis of the patients was analysed with a 24-month survival rate. High PD-ECGF, > 2500 pg $mg^{-1}$ protein, $n$ = 24; low PD-ECGF, < 1000 pg $mg^{-1}$ protein, $n$ = 7

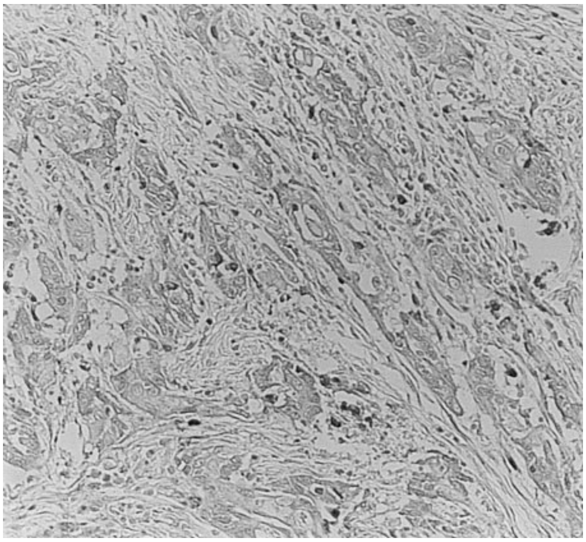

**Figure 2** Immunohistochemical staining for PD-ECGF in uterine cervical cancer. Positive staining is seen in the cytoplasm and nuclear compartments of the cancer cells and in the interstitium (original magnification × 200)

Five cycles of PCR for PD-ECGF mRNA, consisting of denaturation for 1 min at 94°C, annealing for 1 min at 55°C, and extension for 1 min at 72°C, were carried out with reverse transcribed cDNA, 0.1 μM specific primers and Vent DNA polymerase (New England Biolabs, Beverly, MA, USA) in a buffer of 10 mM potassium chloride, 20 mM tris-HCl, pH 8.8, 10 mM diammonium sulphate, 2 mM magnesium sulphate, 0.1% Triton X-100 and 0.15 mM deoxynucleotide phosphates using the IWAKI thermal sequencer TSR-300 (Iwaki Glass, Tokyo, Japan). Additionally, 23 cycles of PCR for PD-ECGF and glyceraldehyde-3-phosphate dehydrogenase (GAPDH) mRNA as an internal standard were carried out in the same manner.

The oligodeoxynucleotides of specific primers in PCR were synthesized according to the published information on cDNA for PD-ECGF (Hagiwara et al, 1991) and GAPDH (Arcari et al, 1984) as follows: sense primer for PD-ECGF mRNA: 5′-AGTCGGATG-GCCATCAGCAT-3′ (in exon 2); antisense primer for PD-ECGF mRNA: 5′-TGGAATGCTTGTCCACAAGC-3′ (in exon 3); sense primer for GAPDH mRNA: 5′-TGAAGGTCGGAGTCAACG-GATTTGGT-3′ (in exon 2); antisense primer for GAPDH mRNA: 5′-CATGTGGGCCATGAGGTCCACCAC-3′ (in exon 8).

 *© Cancer Research Campaign 1999*

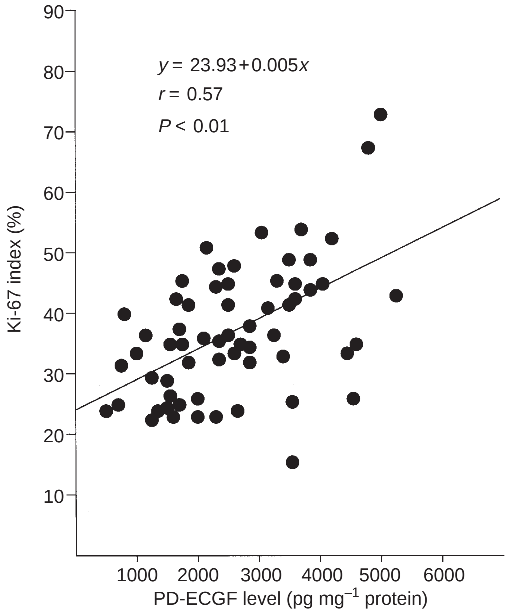

**Figure 3** Correlation between PD-ECGF level and Ki-67 index

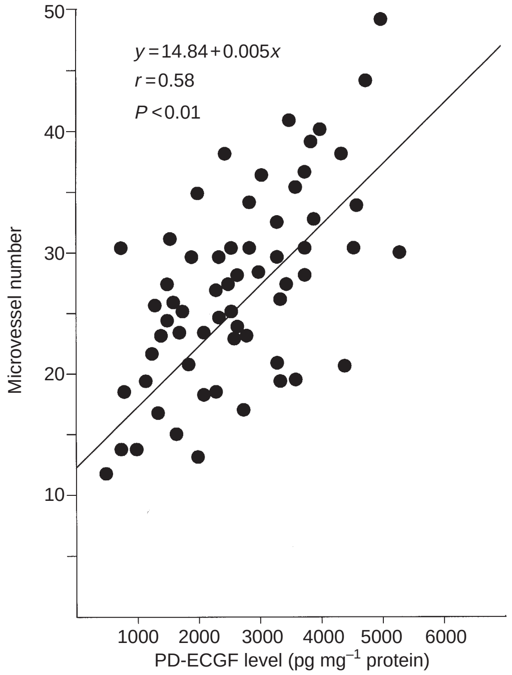

**Figure 4** Correlation between PD-ECGF level and microvessel count

### Southern blot analysis for quantities of PD-ECGF mRNA expression

PCR products were applied to 1.2% agarose gel, and electrophoresis was performed at 50–100 V. PCR products were capillary transferred to an Immobilon transfer membrane (Millipore, Bedford, MA, USA) for 16 h. The membrane was dried at 80°C for 30 min, and was UV irradiated to tightly fix the PCR products. PCR products on the membrane were prehybridized in 1 M sodium chloride, 50 mM Tris-HCl, pH 7.6, and 1% sodium dodecyl sulphate at 42°C for 1 h, and hybridized in the same solution with the biotinylated oligodeoxynucleotide probes synthesized from the sequences of PD-ECGF and GAPDH cDNAs between the specific primers at 65°C overnight. Specific bands hybridized with the biotinylated probes were detected with Plex Luminescent Kits (Millipore), and radiographic film was exposed on the membrane at room temperature for 10 min. The quantification of Southern blot was carried out with Bio Image (Millipore, Ann Arbor, MI, USA). The intensity of specific bands was standardized with that of GAPDH mRNA.

### Statistics

Survival curves were calculated using the Kaplan–Meier method and analysed with the log-rank test. The correlations between the level of PD-ECGF and microvessel count, and between the level of PD-ECGF and Ki-67 index were analysed with Spearman's correlation coefficient. The levels of PD-ECGF and its mRNA were measured from three parts of the same tissue in triplicate. Statistical analysis was performed with Student's *t*-test. Differences were considered significant when the *P*-value was less than 0.05.

### RESULTS

Among the 62 patients who underwent curative resection and were observed for a 24-month survival rate, the prognosis of the 24 patients with high PD-ECGF (> 2500 pg $mg^{-1}$ protein) squamous cell carcinomas was significantly ($P < 0.01$) worse than that of the seven patients with low PD-ECGF (< 1000 pg $mg^{-1}$ protein) squamous cell carcinomas (Figure 1). There was no correlation between PD-ECGF level and the patients' age (data not shown).

In the corresponding 62 tumours, immunohistochemical staining for PD-ECGF was carried out to study PD-ECGF localization in the tumours, and strength of staining was correlated with PD-ECGF levels measured by an enzyme immunoassay. As shown in Figure 2, PD-ECGF was distributed in the surrounding interstitium near cancer cells and in the cytoplasm and nuclear compartments of the cancer cells. PD-ECGF levels correlated approximately with the strength of PD-ECGF staining.

There was a significant correlation between PD-ECGF levels and Ki-67 indices as shown in Figure 3 ($y = 23.93 + 0.005x$, $r = 0.57$, $P < 0.01$), and between PD-ECGF levels and microvessel counts as shown in Figure 4 ($y = 14.84 + 0.005x$, $r = 0.58$, $P < 0.01$). Venous invasion occurred significantly more ($P < 0.01$) in high PD-ECGF tumours than in low PD-ECGF tumours (Figure 5).

The signal intensity curve for mRNA expression was necessary for accurate measurement of the mRNA by RT-PCR. PCR template was prepared from reverse transcribed total RNA (100 μg) from normal uterine cervices as follows: 0.25×, 0.75 μg total RNA reverse transcribed (RNA-RT); 0.5×, 1.5 μg RNA-RT; 1×, 3 μg RNA-RT; 2×, 6 μg RNA-RT; 4×, 12 μg RNA-RT; 8×, 24 μg RNA-RT; and 16×, 48 μg RNA-RT. PCR Southern blot was carried out as described in the Materials and methods section. The signal intensity curve for PD-ECGF mRNA levels ranging from 0.25× to 8× of reverse transcribed total RNA of normal uterine cervices by RT-PCR Southern blot was linear (Figure 6). Therefore, semiquantitative alteration of the mRNA levels was thought to be reliable.

*© Cancer Research Campaign 1999*

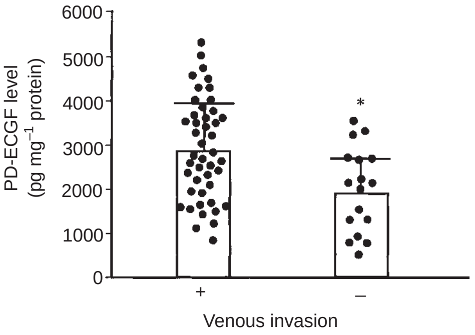

**Figure 5** Correlation between PD-ECGF level and venous invasion. **P* < 0.05 vs positive venous invasion

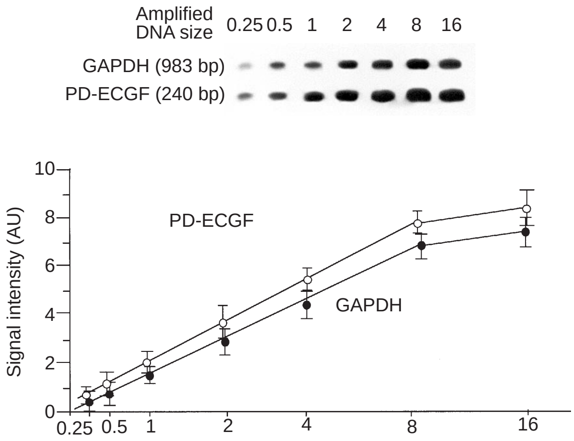

**Figure 6** Signal intensity curve for PD-ECGF mRNA level in a series of reverse transcribed total RNA of normal uterine cervix by reverse transcription polymerase chain reaction (RT-PCR) Southern blot analysis. PCR templates were prepared from reverse transcribed total RNA (100 μg) in normal uterine cervices as follows: 0.25 ×, 0.75 μg total RNA reverse transcribed (RNA-RT); 0.5 ×, 1.5 μg RNA-RT; 1 ×, 3 μg RNA-RT; 2×, 6 μg RNA-RT; 4×, 12 μg RNA-RT; 8×, 24 μg RNA-RT; and 16×, 48 μg RNA-RT. PCR Southern blot was carried out as described in the Materials and methods section. The levels of mRNA expression in normal uterine cervices were assigned as arbitrary units/GAPDH mRNA (AU/GAPDH mRNA). Data are the means ± S.D. of six determinations

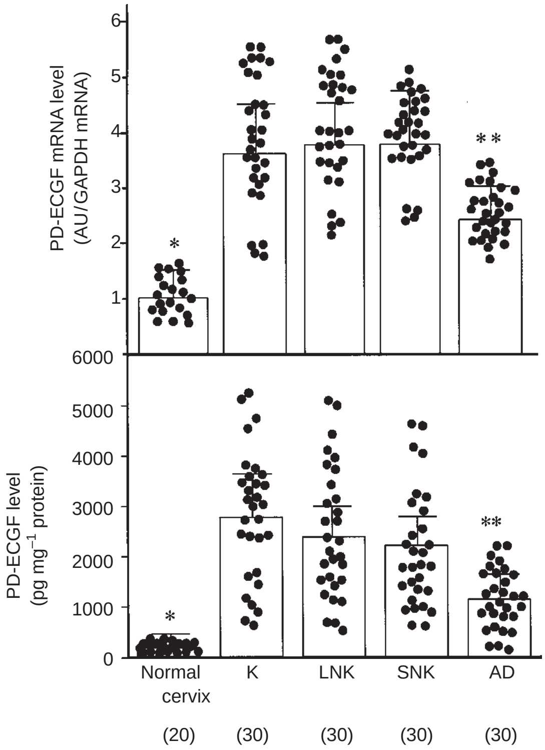

**Figure 7** Levels of PD-ECGF and its mRNA in uterine cervical cancers classified according to histological types. The levels of PD-ECGF and its mRNA were determined by a sandwich enzyme immunoassay and RT-PCR Southern blot analysis respectively. The mRNA level in normal uterine cervices as controls was assigned as AU/GAPDH mRNA. Histological types of uterine cervical cancers are according to the International Federation of Gynaecology and Obstetrics (FIGO) classification. Each level is the mean ± s.d. of nine determinations. K, keratinizing squamous cell carcinoma; LNK, large-cell non-keratinizing squamous cell carcinoma; SNK, small-cell non-keratinizing squamous cell carcinoma; AD, adenocarcinoma. **P* < 0.05 vs K, LNK, SNK and AD; ***P* < 0.1 vs K, LNK and SNK

PD-ECGF and its mRNA had a wider range and were expressed significantly higher ($P < 0.05$) in uterine cervical cancers, especially squamous cell carcinoma, than in normal uterine cervices, regardless of clinical stage (Figures 7 and 8). Furthermore, the levels tended to be higher in squamous cell carcinomas than in adenocarcinomas (Figures 7 and 8).

### DISCUSSION

Newly developed capillary network formation from the original vessel is designated as neovascularization. Generally, turnover of capillary endothelial cells is extremely slow, in the order of months or years in physiological neovascularization, whereas the turnover in ovary and uterine endometrium is rapidly altered along with the ovarian cycle. The turnover with malignant transformation becomes rapid, which might contribute to the acceleration of tumour growth (Denekamp, 1984).

Expression of tumour cell-derived angiogenic factors, basic FGF, VEGF, PD-ECGF and IL-8, may be specific for each tumour and be dependent on the process of tumour growth and spreading. For example, bladder cancers express VEGF in a three-fold excess and PD-ECGF in a 40-fold excess to the corresponding normal tissue (O'Brien et al, 1995). Furthermore, VEGF is dominantly expressed in superficially invasive bladder cancer cases whereas PD-ECGF is dominantly expressed in deeply invasive cases (O'Brien et al, 1995), indicating that the latter is a tumour advancing factor which acts mainly via angiogenic activity. The expression of basic FGF is high in uterine cervical cancers, and increases with dedifferentiation and advancing stage (Fujimoto et al, 1995). In contrast, the expression of VEGF is high in uterine cervical cancers, especially adenocarcinoma, and decreases with advancing stage (data not yet published).

Many authors have reported that a high microvessel density correlates with poor patient prognosis in uterine cervical cancers (Kainz et al, 1995; Rutgers et al, 1995; Wiggins et al, 1995; Bremer et al, 1996; Dinh et al, 1996; Dellas et al, 1997; Obermair et al, 1998). In the present study, the prognosis of the patients with high PD-ECGF squamous cell carcinomas was worse than those with low PD-ECGF squamous cell carcinomas, and PD-ECGF expression correlated with Ki-67 index as an indicator of cellular proliferation (Sawhney and Hall, 1992), microvessel density and

*© Cancer Research Campaign 1999*

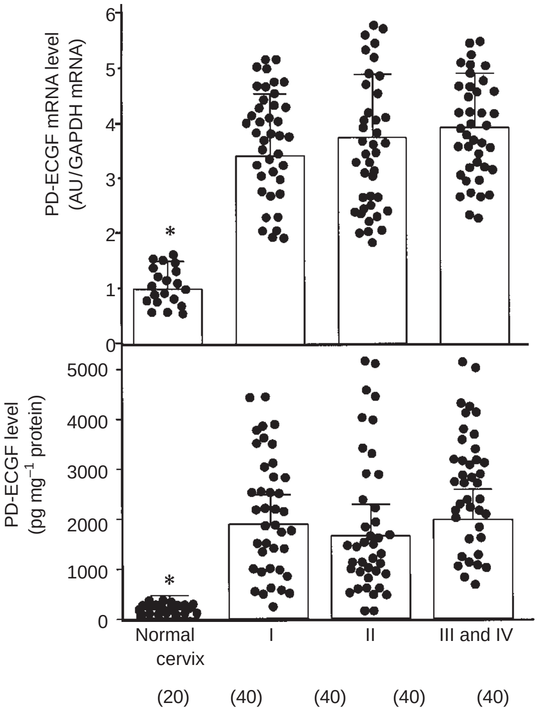

**Figure 8** Levels of PD-ECGF and its mRNA in uterine cervical cancers classified according to clinical stages. Clinical stages of uterine cervical cancer are according to FIGO. *$P < 0.05$ vs I, II, and III and IV

vascular invasion. Therefore, PD-ECGF might contribute to the growth of uterine cervical cancers via angiogenesis related to vascular spreading. Furthermore, the levels of PD-ECGF and its mRNA were higher in uterine cervical cancers, especially squamous cell carcinomas, than in normal uterine cervices, however they did not alter with different histopathological types among squamous cell carcinomas or with advancing stage. In immunohistochemical studies, stronger staining of PD-ECGF is found in squamous cell carcinomas than in adenocarcinomas of the uterine cervix (Tokumo et al, 1998). The tumour cell-derived angiogenic factors basic FGF, VEGF and PD-ECGF may be uniquely expressed and dependent on the process of tumour growth and spreading, and PD-ECGF in uterine cervical cancers might play a role of basic angiogenesis in all processes of advancing of uterine cervical cancers. This indicates that 5′-deoxy-5-fluorouridine might be highly effective on squamous cell carcinomas of the uterine cervix, which possesses a high activity of thymidine phosphorylase to convert 5′-deoxy-5-fluorouridine to 5-fluorouracil (Miwa et al, 1987) regardless of the clinical stage, but related to patients' prognosis, and that some angiogenic inhibitors (Ingber et al, 1990) of new capillary formation might be effective in the inhibition of tumour growth and spreading associated with angiogenesis regardless of a direct tumoral effect on cancer cells.

### REFERENCES

Arcari P, Martinelli R and Salvatore F (1984) The complete sequences of a full length cDNA for human liver glyceraldehyde-3-phosphate dehydrogenase: evidence for multiple mRNA species. *Nucleic Acids Res* **12**: 9179–9189

Bradford M (1976) A rapid and sensitive method for the quantitation of microgram quantities of protein utilizing the principle of protein-dye binding. *Anal Biochem* **72**: 315–323

Bremer GL, Tiebosch AT, van der Putten HW, Schouten HJ, de Haan J and Arends JW (1996) Tumor angiogenesis: an independent prognostic parameter in cervical cancer. *Am J Obstet Gynecol* **174**: 126–131

Chomczynski P and Sacchi N (1987) Single-step method of RNA isolation by acid guanidium thiocyanate–phenol–chloroform extraction. *Anal Biochem* **162**: 156–159

Dellas A, Moch H, Schultheiss E, Feichter G, Almendral AC, Gudat F and Torhorst J (1997) Angiogenesis in cervical neoplasia: microvessel quantitation in precancerous lesions and invasive carcinoma with clinicopathological correlations. *Gynecol Oncol* **67**: 27–33

Denekamp J (1984) Vascular as a target for tumor therapy. *Prog Appl Microcirc* **4**: 28–38

Dinh TV, Hannigan EV, Smith ER, Hove MJ, Chopra V and To T (1996) Tumor angiogenesis as a predictor of recurrence in stage Ib squamous cell carcinoma of the cervix. *Obstet Gynecol* **87**: 751–754

FIGO News (1989) Cervical cancer staging. *Int J Gynecol Obstet* **28**: 189–193

Folkman J (1985) Tumor angiogenesis. *Adv Cancer Res* **43**: 175–203

Folkman J and Haudenschild C (1980) Angiogenesis in vitro. *Nature* **288**: 551–556

Fujimoto J, Hori M, Ichigo S and Tamaya T (1995) Expression of basic fibroblast growth factor and its mRNA in uterine endometrial cancers. *Invasion Metastasis* **15**: 203–210

Fujimoto J, Ichigo S, Sakaguchi H, Hirose R and Tamaya T (1998) Expression of platelet-derived endothelial cell growth factor and its mRNA in uterine endometrium during the menstrual cycle. *Mol Hum Reprod* **4**: 509–513

Fujimoto J, Sakaguchi H, Ichigo S, Hirose R and Tamaya T (1998) Expression of platelet-derived endothelial cell growth factor (PD-ECGF) and its mRNA in ovarian cancers. *Cancer Lett* **126**: 83–88

Fujimoto J, Sakaguchi H, Ichigo S, Hirose R and Tamaya T (1998) Expression of platelet-derived endothelial cell growth factor (PD-ECGF) and its mRNA in uterine endometrial cancers. *Cancer Lett* **130**: 115–120

Fujimoto J, Sakaguchi H, Hirose R and Tamaya T (1999) Expression of platelet-derived endothelial cell growth factor related to angiogenesis in ovarian endometriosis. *J Clin Endocrinol Metab* (in press)

Furukawa T, Yoshimura A, Sumizawa T, Haraguchi M and Akiyama S (1992) Angiogenic factor. *Nature* **356**: 668

Hagiwara K, Stenman G, Honda H, Sahlin P, Anderson A, Miyazono K, Heldin C-H, Ishikawa F and Takaku F (1991) Organization and chromosomal localization of the human platelet-derived endothelial cell growth factor gene. *Mol Cell Biol* **11**: 2125–2132

Haraguchi M, Miyadara K, Uemura K, Sumizawa T, Furukawa T, Yamada K and Akiyama S (1994) Angiogenic activity of enzymes. *Nature* **368**: 198

Ingber D, Fijita T, Kishimoto S, Sudo K, Kanamaru T, Brem H and Folkman J (1990) Synthetic analogues of fumagillin that inhibit angiogenesis and suppress tumour growth. *Nature* **348**: 555–557

Ishikawa F, Miyazono K, Hellman U, Drexler H, Wernstedt C, Hagiwara K, Usuki K, Takaku F, Risau W and Heldin C-H (1989) Identification of angiogenic activity and the cloning and expression of platelet-derived endothelial cell growth factor. *Nature* **338**: 557–562

Kainz C, Speiser P, Wanner C, Obermair A, Tempfer C, Sliutz G, Reinthaller A and Brettenecker G (1995) Prognostic value of tumour microvessel density in cancer of the uterine cervix stage IB to IIB. *Anticancer Res* **15**: 1549–1551

Macchiarini P, Fontanini G, Hardin MJ, Squartini F and Angeletti GA (1992) Relation of neovascularization to metastasis of non-small-cell lung cancer. *Lancet* **340**: 145–146

Maeda K, Chung YS, Ogawa S, Takatsuka S, Kanng SM, Ogawa M, Sawada T, Onoda N, Kato Y and Sowa M (1996) Thymidine phosphorylase/platelet-derived endothelial cell growth factor associated with hepatic metastasis in gastric carcinoma. *Br J Cancer* **73**: 884–888

Miwa M, Nishimura J, Kamiyama T and Ishitsuka H (1987) Conversion of 5′-deoxyfluorouridine to 5-FU by pyrimidine nucleotide phosphorylases in normal and tumor tissues from rodents bearing tumors and cancer patients. *Jpn J Cancer Chemother* **14**: 2924–2929

Miyadera K, Sumizawa T, Haraguchi M, Yoshida H, Konstanty W, Yamada Y and Akiyama S (1995) Role of thymidine phosphorylase activity in the angiogenic effect of platelet-derived endothelial cell growth factor/thymidine phosphorylase. *Cancer Res* **55**: 1687–1690

Nakano T and Oka K (1993) Differential values of Ki-67 index and mitotic index of proliferating cell population. An assessment of cell cycle and prognosis in radiation therapy for cervical cancer. *Cancer* **72**: 2401–2408

Nishida M, Hino A, Mori K, Matsumoto T, Yoshikubo T and Ishitsuka H (1996) Preparation of anti-human thymidine phosphorylase monoclonal antibodies useful for detecting the enzyme levels in tumor tissues. *Biol Pharm Bull* **19**: 1407–1411

O'Brien T, Cranston D, Fuggle S, Bicknell R and Harris AL (1995) Different angiogenic pathways characterize superficial and invasive bladder cancer. *Cancer Res* **55**: 510–513

Obermair A, Wanner C, Bilgi S, Speiser P, Kaider A, Reinthaller A, Leodolter S and Gitsch G (1998) Tumor angiogenesis in stage IB cervical cancer: correlation of microvessel density with survival. *Am J Obstet Gynecol* **178**: 314–319

Rutgers JL, Mattox TF and Vargas MP (1995) Angiogenesis in uterine cervical squamous cell carcinoma. *Int J Gynecol Pathol* **14**: 114–118

Sawhney N and Hall PA (1992) Ki-67 structure, function and new antibodies. *J Pathol* **168**: 161–162

Srivastava A, Laidler P, Davies RP, Horgan K and Hughes L (1988) The prognostic significance of tumor vascularity in intermediate-thickness (0.76–4.0 mm thick) skin melanoma. *Am J Pathol* **133**: 419–423

Sumizawa T, Furukawa T, Haraguchi M, Yoshimura A, Takeyasu A, Ishizawa M, Yamada Y and Akiyama S (1993) Thymidine phosphorylase activity associated with platelet-derived endothelial cell growth factor. *J Biochem* **114**: 9–14

Tokumo K, Kodama J, Nakanishi Y, Miyagi Y, Kamimura S, Yoshinouchi M, Okuda H and Kudo T (1998) Different angiogenic pathways in human cervical cancers. *Gynecol Oncol* **68**: 38–44

Wakui S, Furusato M, Itoh T, Sasaki H, Akiyama A, Kinoshita I, Asano K, Tokuda T, Aizawa S and Ushigome S (1992) Tumor angiogenesis in prostatic carcinoma with and without bone marrow metastasis: a morphometric study. *J Pathol* **168**: 257–262

Weidner N, Semple JP, Welch WR and Folkman J (1991) Tumor angiogenesis and metastasis – correlation in invasive breast carcinoma. *N Engl J Med* **324**: 1–7

Weidner N, Carroll PR, Flax J, Blumenfeld W and Folkman J (1993) Tumor angiogenesis correlates with metastasis in invasive prostate carcinoma. *Am J Pathol* **143**: 401–409

Wiggins DL, Granaico, Steinhoff MM and Calabresi P (1995) Tumor angiogenesis as a prognostic factor in cervical carcinoma. *Gynecol Oncol* **56**: 353–356

Yoshimura A, Kuwazuru Y, Furukawa T, Yoshida H, Yamada K and Akiyama S (1990) Purification and tissue distribution of human thymidine phosphorylase; high expression in lymphocytes, reticulocytes and tumors. *Biochem Biophys Acta* **1034**: 107–113

*© Cancer Research Campaign 1999*

## References

[PDF_00411] Arcari P., Martinelli R., Salvatore F. (1984). The complete sequence of a full length cDNA for human liver glyceraldehyde-3-phosphate dehydrogenase: evidence for multiple mRNA species.. Nucleic Acids Res.

[PDF_00417] Bremer G. L., Tiebosch A. T., van der Putten H. W., Schouten H. J., de Haan J., Arends J. W. (1996). Tumor angiogenesis: an independent prognostic parameter in cervical cancer.. Am J Obstet Gynecol.

[PDF_00414] Burdett P. E., Kipps A. E., Whitehead P. H. (1976). A rapid technique for the detection of amylase isoenzymes using an enzyme sensitive "test-paper".. Anal Biochem.

[PDF_00420] Chomczynski P., Sacchi N. (1987). Single-step method of RNA isolation by acid guanidinium thiocyanate-phenol-chloroform extraction.. Anal Biochem.

[PDF_00429] Dinh T. V., Hannigan E. V., Smith E. R., Hove M. J., Chopra V., To T. (1996). Tumor angiogenesis as a predictor of recurrence in stage Ib squamous cell carcinoma of the cervix.. Obstet Gynecol.

[PDF_00434] Folkman J., Haudenschild C. (1980). Angiogenesis in vitro.. Nature.

[PDF_00433] Folkman J. (1985). Tumor angiogenesis.. Adv Cancer Res.

[PDF_00435] Fujimoto J., Hori M., Ichigo S., Tamaya T. (1995). Expression of basic fibroblast growth factor and its mRNA in uterine endometrial cancers.. Invasion Metastasis.

[PDF_00444] Fujimoto J., Ichigo S., Sakaguchi H., Hirose R., Tamaya T. (1998). Expression of platelet-derived endothelial cell growth factor (PD-ECGF) and its mRNA in uterine endometrial cancers.. Cancer Lett.

[PDF_00438] Fujimoto J., Ichigo S., Sakaguchi H., Hirose R., Tamaya T. (1998). Expression of platelet-derived endothelial cell growth factor and its mRNA in uterine endometrium during the menstrual cycle.. Mol Hum Reprod.

[PDF_00452] Hagiwara K., Stenman G., Honda H., Sahlin P., Andersson A., Miyazono K., Heldin C. H., Ishikawa F., Takaku F. (1991). Organization and chromosomal localization of the human platelet-derived endothelial cell growth factor gene.. Mol Cell Biol.

[PDF_00456] Haraguchi M., Miyadera K., Uemura K., Sumizawa T., Furukawa T., Yamada K., Akiyama S., Yamada Y. (1994). Angiogenic activity of enzymes.. Nature.

[PDF_00458] Ingber D., Fujita T., Kishimoto S., Sudo K., Kanamaru T., Brem H., Folkman J. (1990). Synthetic analogues of fumagillin that inhibit angiogenesis and suppress tumour growth.. Nature.

[PDF_00461] Ishikawa F., Miyazono K., Hellman U., Drexler H., Wernstedt C., Hagiwara K., Usuki K., Takaku F., Risau W., Heldin C. H. (1989). Identification of angiogenic activity and the cloning and expression of platelet-derived endothelial cell growth factor.. Nature.

[PDF_00465] Kainz C., Speiser P., Wanner C., Obermair A., Tempfer C., Sliutz G., Reinthaller A., Breitenecker G. (1995). Prognostic value of tumour microvessel density in cancer of the uterine cervix stage IB to IIB.. Anticancer Res.

[PDF_00469] Macchiarini P., Fontanini G., Hardin M. J., Squartini F., Angeletti C. A. (1992). Relation of neovascularisation to metastasis of non-small-cell lung cancer.. Lancet.

[PDF_00472] Maeda K., Chung Y. S., Ogawa Y., Takatsuka S., Kang S. M., Ogawa M., Sawada T., Onoda N., Kato Y., Sowa M. (1996). Thymidine phosphorylase/platelet-derived endothelial cell growth factor expression associated with hepatic metastasis in gastric carcinoma.. Br J Cancer.

[PDF_00480] Miyadera K., Sumizawa T., Haraguchi M., Yoshida H., Konstanty W., Yamada Y., Akiyama S. (1995). Role of thymidine phosphorylase activity in the angiogenic effect of platelet derived endothelial cell growth factor/thymidine phosphorylase.. Cancer Res.

[PDF_00484] Nakano T., Oka K. (1993). Differential values of Ki-67 index and mitotic index of proliferating cell population. An assessment of cell cycle and prognosis in radiation therapy for cervical cancer.. Cancer.

[PDF_00487] Nishida M., Hino A., Mori K., Matsumoto T., Yoshikubo T., Ishitsuka H. (1996). Preparation of anti-human thymidine phosphorylase monoclonal antibodies useful for detecting the enzyme levels in tumor tissues.. Biol Pharm Bull.

[PDF_00520] O'Brien T., Cranston D., Fuggle S., Bicknell R., Harris A. L. (1995). Different angiogenic pathways characterize superficial and invasive bladder cancer.. Cancer Res.

[PDF_00523] Obermair A., Wanner C., Bilgi S., Speiser P., Kaider A., Reinthaller A., Leodolter S., Gitsch G. (1998). Tumor angiogenesis in stage IB cervical cancer: correlation of microvessel density with survival.. Am J Obstet Gynecol.

[PDF_00527] Rutgers J. L., Mattox T. F., Vargas M. P. (1995). Angiogenesis in uterine cervical squamous cell carcinoma.. Int J Gynecol Pathol.

[PDF_00529] Sawhney N., Hall P. A. (1992). Ki67--structure, function, and new antibodies.. J Pathol.

[PDF_00531] Srivastava A., Laidler P., Davies R. P., Horgan K., Hughes L. E. (1988). The prognostic significance of tumor vascularity in intermediate-thickness (0.76-4.0 mm thick) skin melanoma. A quantitative histologic study.. Am J Pathol.

[PDF_00541] Wakui S., Furusato M., Itoh T., Sasaki H., Akiyama A., Kinoshita I., Asano K., Tokuda T., Aizawa S., Ushigome S. (1992). Tumour angiogenesis in prostatic carcinoma with and without bone marrow metastasis: a morphometric study.. J Pathol.

[PDF_00547] Weidner N., Carroll P. R., Flax J., Blumenfeld W., Folkman J. (1993). Tumor angiogenesis correlates with metastasis in invasive prostate carcinoma.. Am J Pathol.

[PDF_00552] Yoshimura A., Kuwazuru Y., Furukawa T., Yoshida H., Yamada K., Akiyama S. (1990). Purification and tissue distribution of human thymidine phosphorylase; high expression in lymphocytes, reticulocytes and tumors.. Biochim Biophys Acta.

